# Endovascular Treatment of Complex Vascular Diseases of the Internal Carotid Artery Using the Willis Covered Stent: Preliminary Experience and Technical Considerations

**DOI:** 10.3389/fneur.2020.554988

**Published:** 2020-10-29

**Authors:** Lin Ma, Hao Feng, Shuo Yan, Ji-Chong Xu, Hua-Qiao Tan, Chun Fang

**Affiliations:** Department of Interventional Radiology, Shanghai Tongji Hospital, Tongji University School of Medicine, Shanghai, China

**Keywords:** endovascular treatment, covered stent, internal carotid artery, aneurysm, endoleak

## Abstract

**Purpose:** The Willis covered stent (WCS) is used to treat complex vascular diseases of the internal carotid artery; however, its performance requires further investigation. This study aimed to present our single-center clinical results and experience of endovascular repair of complex vascular diseases of the internal carotid artery using the WCS.

**Methods:** Patients who presented with complex vascular diseases of the internal carotid artery and who were treated with the WCS from December 2013 to September 2018 were retrospectively reviewed. Procedural results, perioperative complications, incidence of endoleak, and follow-up outcomes were analyzed.

**Results:** Sixty-five patients were enrolled. A total of 25 large aneurysms, 10 pseudoaneurysms, 14 blood blister-like aneurysms, 11 carotid–cavernous fistulas, and 5 surgical injuries were assessed. WCS placement was successful in all patients. Immediate angiography showed that complete repair of the target artery was achieved in 56 patients (86.2%). Endoleak was observed in nine patients, including seven type I endoleaks and two type II endoleaks. Occlusion of a side-branch vessel occurred in four patients. Acute in-stent thrombosis occurred in one patient. No ischemic or hemorrhagic events or other complications developed during the perioperative and follow-up periods. Angiographic follow-up (mean duration, 12 ± 3.29 months) was performed in 60 patients and showed complete target artery repair in 58 patients, and asymptomatic mild to moderate in-stent stenosis was observed in four patients. Slight endoleak persisted in two patients without enlargement or rupture of the residual lumen.

**Conclusion:** WCS implantation is safe, feasible, and efficacious for endovascular repair in patients with complex vascular diseases of the internal carotid artery, showing excellent short-term target artery patency and clinical outcomes.

## Introduction

Complex vascular diseases of the internal carotid artery (ICA) are associated with a poor response and high complication rates when treated with conventional endovascular or surgical treatments. This is thought to be due to the affected anatomical structures and the morphological and histological features of these diseases. Such diseases include large aneurysms, blood blister-like aneurysms (BBAs), and carotid–cavernous fistulas (CCFs).

At present, commonly used treatments for vascular diseases of the ICA include endovascular treatment and surgery. Endovascular treatment methods include coil embolization, stent-assisted coiling, detachable balloon embolization, and flow diverter stenting (FDS) (1–4). However, high rates of incomplete occlusion and recanalization have been reported in the treatment of large aneurysms. High rates of treatment-related complications have also been observed in the treatment of BBAs, and high rates of pseudoaneurysm formation and incomplete occlusion have been observed in the treatment of CCFs (3–5). The application of frequently used surgical strategies, including vessel ligation, clipping, wrapping, trapping, and extracranial–intracranial bypass, is limited by bony obstacles, adjacent vital anatomical structures of ICA, severe intraoperative bleeding, and postoperative regrowth rates (6–11).

Because of the drawbacks of routine treatments for complex vascular diseases of the ICA, innovative techniques and products are required. Recently, the Willis covered stent (WCS; MicroPort, Shanghai, China), which was designed for the intracranial vasculature, has become a promising option for the treatment of complex vascular diseases of the ICA. The WCS consists of a bare metal stent, an expandable polytetrafluoroethylene membrane covering the outside of the stent, and a rapid exchange balloon system. The specific parameters of the device and placement method have been described previously (12). The potential mechanisms of the WCS include immediate lesion exclusion from the circulation and vessel wall reconstruction.

Since its launch in China, the WCS has been widely used to treat various complicated intracranial ICA diseases and has been gradually introduced to treat extracranial ICA and vertebral artery diseases, including large aneurysms, BBAs, pseudoaneurysms, and CCFs, with promising feasibility and efficacy (13–17). Despite these encouraging results, there are insufficient data on the safety, feasibility, and efficacy of endovascular treatment of complex vascular diseases of the ICA using the WCS. In this study, we report our clinical results and experience using a retrospective analysis of 65 patients with complex vascular diseases of the ICA treated by WCS placement. To our knowledge, this study is the largest series reported on the clinical and angiographic outcomes of WCS placement for the treatment of complex vascular diseases of the ICA.

## Materials and Methods

### Participants

Between December 2013 and September 2018, patients who presented with complex vascular diseases of the ICA and who were treated with WCS implantation at our institution were enrolled. The inclusion criteria were as follows: (1) the presence of a large aneurysm (>10 mm), traumatic or radiation-induced pseudoaneurysm, BBA, CCF, and surgical injury, located in the ICA, which was likely to be difficult to treat or presented an high risk if it was treated by conventional coil procedures or surgical treatments; (2) the modified Rankin Scale (mRS) score ≤ or Hunt–Hess grade ≤ III; (3) the patient or family member willing to sign informed consent before undergoing endovascular procedures and clinical data collection. Exclusion criteria were as follows: (1) a distance of <2 mm between the aneurysm orifice and the anterior choroidal artery, fetal-type posterior communicating artery; or (2) an extremely tortuous parent artery that prohibited navigation and placement of the WCS delivery system; or (3) known allergy or contraindication to aspirin and clopidogrel, aspirin, and clopidogrel resistance or intolerance of general anesthesia; or (4) the prognosis predicted to be very poor.

Clinical data, technical results, and follow-up outcomes were analyzed. Some cases including some large aneurysms and BBAs have been reported in our previous studies (14, 18). This study was approved by our institutional review board committee. The ICA classification system used in this study was in accordance with the description outlined by Bouthillier et al. in 1996 (19).

### Endovascular Treatment Procedures

**Figure 1:**
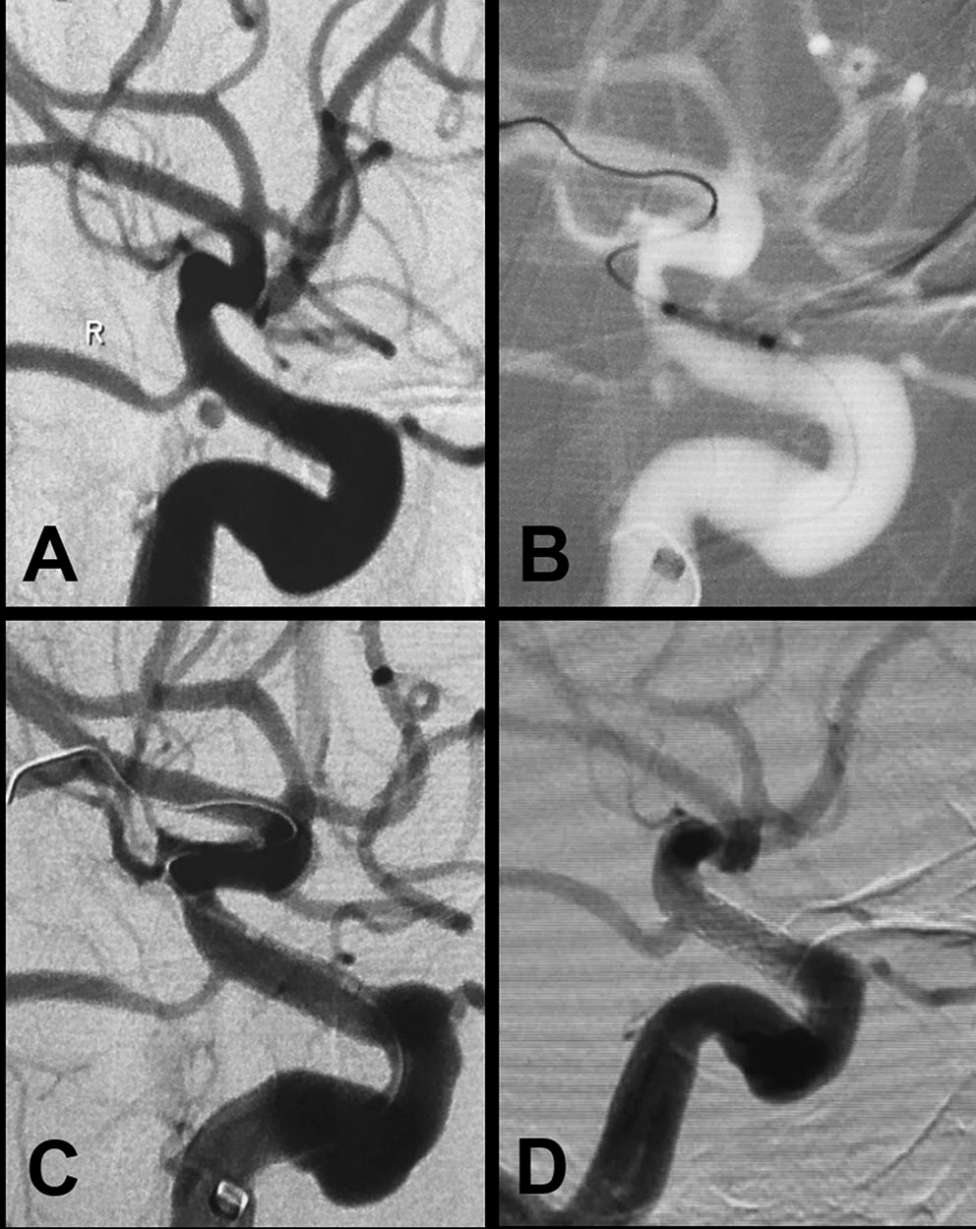
A blood blister-like aneurysm at the ophthalmic segment of the ICA treated with the Willis covered stent. **(A)** Preprocedural digital subtraction angiography reveals a blood blister-like aneurysm (2.5 × 1.6 mm) in the posterior wall of the ophthalmic segment of the ICA. **(B)** The Willis covered stent (3.5 × 7 mm) was precisely delivered to the location of the aneurysm under roadmap-mask guidance. **(C)** Cerebral angiography immediately after stent placement demonstrates complete occlusion of the aneurysm with parent artery patency. **(D)** Twelve-month follow-up digital subtraction angiography shows complete obliteration of the aneurysm with no parent artery stenosis.

All procedures were performed under general anesthesia. A 6-Fr long sheath (Cook, Bloomington, USA) was initially positioned in the cervical segment of the ICA. A 6-Fr Neuron (Penumbra, Alameda, California, USA) or Navien (Ev3/Covidien, California, USA) intermediate support catheter was advanced approximate to the target lesion. A 300- or 205-cm-long and 0.014-inch-diameter micro-guidewire (Transcend, Boston Scientific, California, USA) was navigated into the distal segment of the parent artery. The WCS is available in various diameters (range, 3.5–4.5 mm) and lengths (range, 7–16 mm). The selected stent should be at most 0.5 mm wider in diameter than the target artery and at least 4 mm longer than the lesion neck. Under guidance of the roadmap, the stent was advanced over the micro-guidewire to bridge the lesion orifice. Multiple control angiograms were obtained to confirm the position of the stent and avoid covering the important side branch. Then, the stent was deployed using 5- to 6-atm pressure. Angiography was performed immediately after balloon deflation to confirm correct stent placement and satisfactory lesion occlusion (Figure 1).

**Figure 2:**
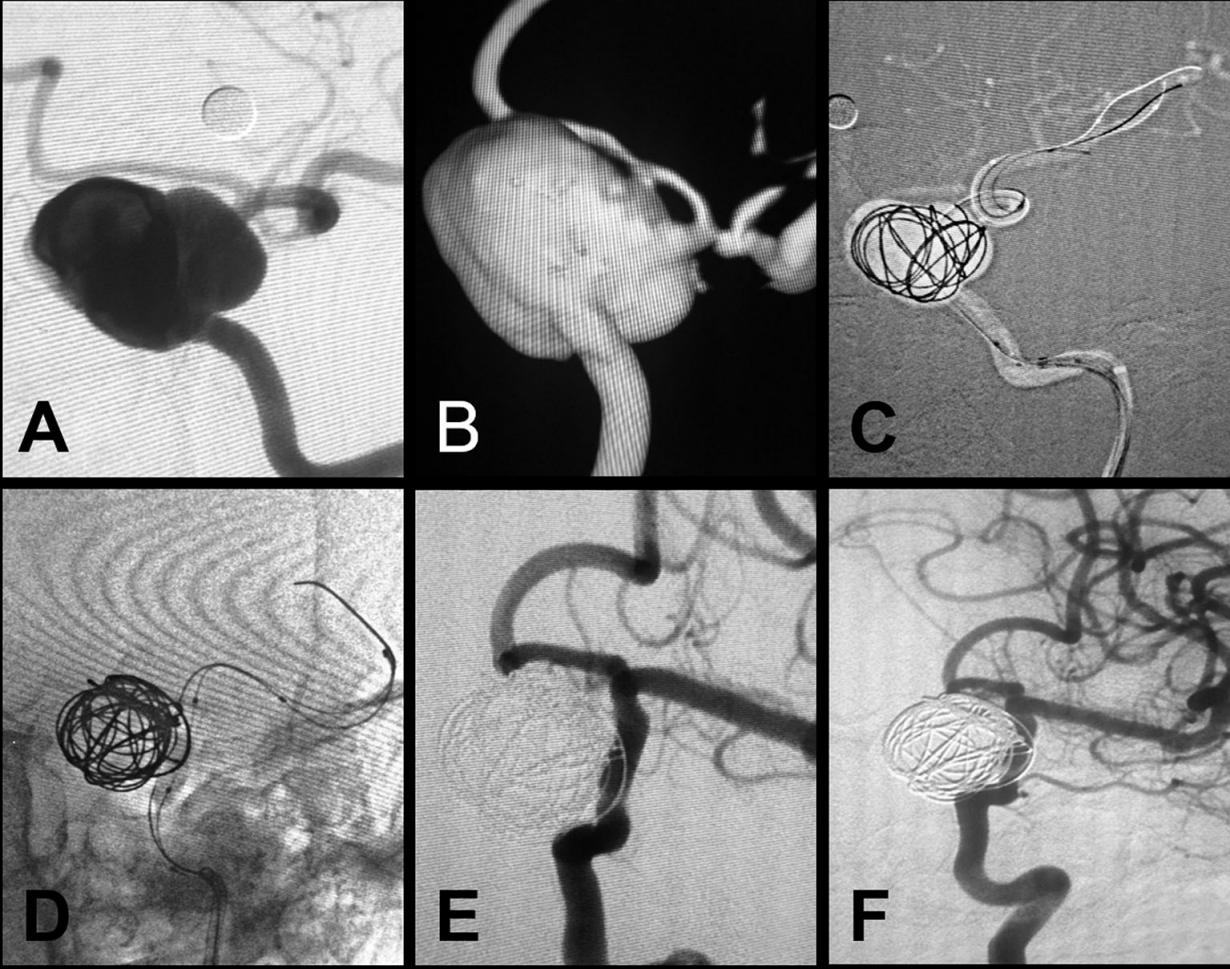
A large aneurysm in the clinoid and ophthalmic segment of the internal carotid artery treated with the Willis covered stent plus coil embolization. **(A,B)** Preprocedural digital subtraction angiography shows a wide-necked (neck width, 10.5 mm) large aneurysm (20 × 26 mm) in the clinoid–supraclinoid segment of the ICA. **(C)** A support catheter (Navien) was positioned in the cavernous segment of the ICA, and a double-microcatheter technique was used for coil embolization (20 × 50 mm). **(D)** The Willis covered stent (3.5 × 16 mm) was successfully deployed with the proximal and distal ends of the stent covering the neck of the aneurysm on both sides, and the Willis covered stent was deployed by balloon inflation. **(E)** Immediate postprocedural angiography shows complete exclusion of the aneurysm with parent artery patency. **(F)** Twelve-month follow-up digital subtraction angiography shows complete obliteration of the aneurysm with parent artery patency.

For large aneurysms (>10 mm) or lesions located in curved segments or closely related to the perforating vessel, before stent deployment, several coils were placed in the cavity through another microcatheter to decrease the incidence of endoleak (Figure 2). If the lesion was located beyond the cavernous segment of the ICA, or if the parent artery was seriously tortuous, the intermediate support catheter was pushed across the segment of the lesion using a coaxial technique. Then, the stent was pushed through the support catheter to the correct position, and the support catheter was pulled back to unsheathe the covered stent, avoiding stent membrane damage during delivery, which is referred to as the intermediate-catheter “Trojan horse” technique.

### Antithrombotic Treatment

Before the procedure, patients with unruptured aneurysms or CCFs were treated with daily doses of 100 mg of aspirin and 75 mg of clopidogrel for at least for 3 days, and then thrombelastography was performed to confirm drug effectiveness. For urgent conditions, such as subarachnoid hemorrhage (SAH) or epistaxis caused by ruptured aneurysms, loading doses of 300 mg of aspirin and 300 mg of clopidogrel were administrated through a nasogastric tube before the procedure. All patients received systemic intravenous heparin during the procedure to maintain an activated clotting time of between 250 and 300 s. After WCS placement, tirofiban was instantly administered through an intermediate catheter to avoid acute thrombosis. After the procedure, tirofiban was administered for 48 h (intravenous infusion at a concentration of 0.15 μg/kg per minute) to avoid in-stent thrombosis in patients whose intracranial ruptured lesions were completely excluded.

Thereafter, all patients were administered a dual antiplatelet regimen for 6–12 months according to the degree of stent endothelialization, and then aspirin or clopidogrel was administered alone for at least 2 years.

### Imaging and Clinical Follow-Up

Imaging follow-up was performed at 3 and 6–12 months after the procedure then annually thereafter. Clinical follow-up was performed to determine neurological deficits and any changes from baseline neurological status that were related to the devices or the procedure. This was assessed at every admission for follow-up angiography or at the outpatient clinic. The mRS score was used to evaluate the clinical state of patients. During follow-up, patients with any deterioration in neurological status were required to come back to the hospital and undergo brain computed tomography or magnetic resonance imaging if necessary. The status of the lesion was assessed by angiography to exclude the possibility of residual endoleak, aneurysmal regrowth, and in-stent stenosis. Data from the initial and final angiographic results and clinical outcomes were retrospectively collected and analyzed by two experienced neuroradiologists (H-Q.T. and C.F.).

### Statistical Analysis

Statistical analysis was performed using the SPSS software package, version 16.0. Data are presented as mean ± standard deviation for continuous variables, median for continuous variables with skewed distributions, or percentages for nominal variables.

## Results

### Baseline Characteristics of Patients

A total of 65 patients (30 male and 35 female patients) were enrolled with an age range of 19 to 75 years (median, 65 years). A total of 25 large aneurysms (>10 mm), 10 pseudoaneurysms, 14 BBAs, 11 CCFs, and 5 surgical injuries were assessed. Of the 10 pseudoaneurysms, six were traumatic and four were radiation-induced. Among all enrolled patients, 22 had SAH caused by spontaneous aneurysmal rupture in 19 cases and pituitary surgery–related hemorrhage in 3 cases, including Hunt–Hess grade I in 7 cases, grade II in 8 cases, and grade III in 7 cases. Twenty-six patients experienced headache, and 11 patients had epistaxis caused by nasopharyngeal carcinoma radiotherapy in 4 cases, traumatic injury in 4 cases, and paranasal sinus surgery–related hemorrhage in 3 cases. Sixteen patients had visual defects and/or diplopia, 8 had tinnitus, 3 had pituitary endocrine abnormalities, and 12 had exophthalmos/conjunctival congestion. Fifteen vascular diseases were located in the Bouthillier lacerum segment, 20 were located in the cavernous segment, 10 were located in the clinoid segment, and 20 were located in the supraclinoid (ophthalmic plus communicating) segment. The demographics and clinical data of patients are summarized in Table 1.

**Table 1:** Clinical variables of the 65 patients with complex vascular diseases of the ICA treated by endovascular repair with the Willis covered stent.

**Variables**	**Values**
Sex	
Male	30
Female	35
Median age (years)	65 (19–75)
Location in the ICA segment	
Supraclinoid	20
Clinoid	10
Cavernous	20
Lacerum	15
Disease category	
Large aneurysms (>10 mm)	25
Pseudoaneurysms	10
Traumatic	6
Radiation-induced	4
Blood blister-like aneurysms	14
CCF	11
Traumatic	9
Spontaneous	2
Surgical injuries	5
Clinical manifestation	
SAH	22
H & H grade	
I	7
II	8
III	7
Epistaxis	11
Traumatic	4
Nasopharyngeal carcinoma radiotherapy	4
Iatrogenic	3
Headache	26
Visual defect/diplopia	16
Tinnitus	8
Pituitary dysfunction	3
Exophthalmos/conjunctival congestion	12
Treatment strategy	
Single covered stent	33
Double covered stent	2
Single covered stent plus coils	25
Double covered stent plus coils	4
Outcome	
Complete occlusion	56 (86.2%)
Endoleak	9 (13.8%)
Endoleak type	
Type I	7
Type II	2
Endoleak causes	
Poor stent adherence	3
Insufficient overlap	2
Backflow from the branch vessel	2
Adverse events	
Acute stent thrombosis	1
Occlusion of side branch vessel	4
Ophthalmic artery	2
Anterior choroidal artery	1
Posterior communicating artery	1
Follow-up	
Mean time (months)	12 ± 3.29
Angiographic follow-up	60
Complete occlusion	58 (96.7%)
Endoleak	2 (3.3%)
In-stent stenosis	4
Clinical follow-up	65
mRS	
0–1	55
2	10

*ICA, internal carotid artery; SAH, subarachnoid hemorrhage; H&H, Hunt & Hess; CCF, carotid–cavernous sinus fistula; mRS, modified Rankin Scale*.

### Primary Procedural Results

**Figure 3:**
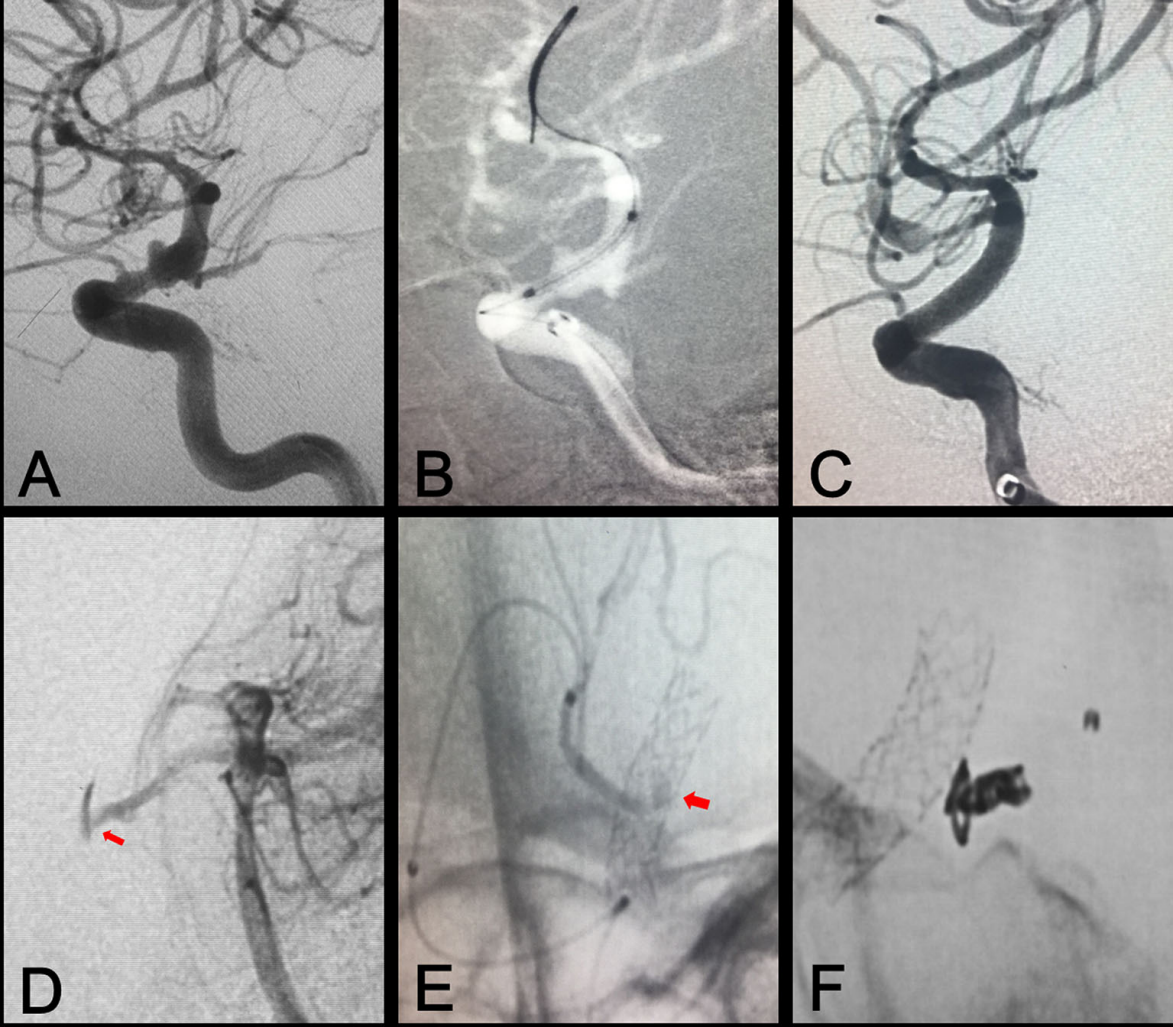
Branch artery embolization for type II endoleak. **(A)** Preprocedural digital subtraction angiography shows a blood blister-like aneurysm in the ophthalmic segment of the ICA. **(B)** A Willis covered stent was positioned at the location of the aneurysm. **(C)** Immediate postprocedural angiography shows complete exclusion of the aneurysm. **(D,E)** Vertebral arteriography shows contrast agent on the outside of the stent, representing type II endoleak resulting from backflow from the posterior communicating artery (arrows). **(F)** Embolization of the posterior communicating artery with coil embolization.

Primary procedural results of all patients are summarized in Tables 1, 2. Deployment of the WCS was technically successful in all patients. Among the 65 patients, 33 patients underwent single-stent implantation, 2 patients underwent double-stent implantation, 26 patients underwent single-stent plus coil embolization, and 4 patients underwent double-stent plus coil embolization. Total isolation of complex vascular diseases of the ICA was achieved immediately in 56 patients (86.2%), and endoleak was observed in 9 patients (type I in 7 patients and type II in 2 patients), including obvious endoleak in 6 patients and slight endoleak in 3 patients (intraluminal retention of contrast media), as summarized in Table 3. Five patients with obvious type I endoleak underwent repeat balloon dilation first, and then 4 patients with sustained endoleak underwent implantation of a second WCS. For two patients with type II endoleak, one with slight endoleak underwent follow-up observation, and the other underwent branch artery embolization (Figure 3). All patients with slight endoleak underwent follow-up observation. Side-branch vessel occlusion occurred in four patients, including two cases of ophthalmic artery occlusion, one case of anterior choroidal artery occlusion, and case of one posterior communicating artery occlusion. Fortunately, no patients showed any clinical symptoms. Acute in-stent thrombosis occurred in one patient; thus, intra-arterial tirofiban was administered, which dissolved the thrombus immediately. No other complications, such as aneurysmal rupture, vessel dissection, or stent displacement, occurred during the procedure.

**Table 2:** Summary of treatment, outcome, and follow-up data for 65 patients with complex vascular diseases of the ICA.

**Disease category**	**Cases**	**Treatment strategy**				**Immediate angiography**		**Follow-up**	**Follow-up angiography**		
		**Single**	**Double**	**Single WCS**	**Double WCS**	**Occlusion**	**Endoleak**	**(12 ± 3.29**	**Occlusion**	**Endoleak**	**In-stent**
		**WCS**	**WCS**	**plus coils**	**plus coils**			**months)**			**stenosis**
Large aneurysm	25	4	1	18	2	20	5	23	22	1	2
Pseudoaneurysm	10	8	0	2	0	10	0	9	9	0	0
Blood blister-like aneurysm	14	14	0	0	0	11	3	13	13	0	2
Carotid–cavernous fistula	11	4	1	6	0	10	1	10	9	1	0
Surgical injury	5	3	0	0	2	5	0	5	5	0	0
Total	65	33	2	26	4	56 (86.2%)	9 (13.8%)	60 (92.3%)	58 (96.7%)	2 (3.3%)	4 (6.7%)

**Table 3:** Endovascular treatment and angiographic follow-up results of 9 patients with endoleak.

**Case No**.	**Age**	**Disease**	**Disease status**		**Treatment**	**Endoleak**	**Endoleak**	**Outcome**	**Follow-up**	
	**(years)**	**category**	**Size**	**Location**	**strategy**	**type**	**treatment**		**Time**	**Endoleak**
			**(mm)**						**(months)**	**outcome**
1	53	BBA/SAH	2.0 × 3.0	Supraclinoid	WCS	Type I/D	Observation	–	12	None
2	39	LAN	10.5 × 9.0	Clinoid	WCS+Coils	Type I/D	Observation	–	18	None
3	43	CCF	–	Clinoid	WCS	Type I/P	BD + WCS	Diminished	13	Slight
4	67	LAN/SAH	8.0 × 12.0	Supraclinoid	WCS+Coils	Type I/P	BD + WCS	Disappeared	12	None
5	22	LAN	16.0 × 18.0	Lacerum	WCS	Type I/D	BD + WCS	Diminished	13	Slight
6	68	LAN	8.0 × 12.0	Clinoid	WCS+Coils	Type II	Observation	–	8	None
7	43	BBA /SAH	3.0 × 2.0	Supraclinoid	WCS	Type I/D	BD	Disappeared	12	None
8	57	BBA/SAH	2.0 × 2.0	Supraclinoid	WCS	Type II	Branch embolization	Disappeared	10	None
9	47	LAN	18.0 × 20.0	Clinoid	WCS+Coils	Type I/D	BD + WCS	Disappeared	11	None

*BBA, blood blister-like aneurysm; SAH, subarachnoid hemorrhage; LAN, large aneurysm; CCF, carotid–cavernous sinus fistula; WCS, Willis covered stent; Type I/P, blood flow from stent proximal site; Type I/D, blood flow from stent distal site; BD, balloon dilatation*.

### Angiographic and Clinical Follow-Up Results

Follow-up results are presented in Tables 1, 2. The mean follow-up period was 12 ± 3.29 months (range, 6–24 months). Follow-up angiography was performed in 60 patients (92.3%), and complete target artery repair was achieved in 58 patients (96.7%). Three patients achieved elimination of slight spontaneous endoleak, and slight endoleak without enlargement or rupture of residual lumen persisted in two patients. Only four patients showed asymptomatic mild to moderate in-stent stenosis. Telephone follow-up assessments were conducted for the remaining patients. During the follow-up period, no ischemic/hemorrhagic events or deaths occurred. Moreover, patients with side-branch vessel occlusion showed no related clinical symptoms. The mRS score at follow-up was 0–1 in 55 patients and 2 in 10 patients.

## Discussion

In this study, we described our clinical results and experience of endovascular repair of complex vascular diseases of the ICA using the WCS. We also showed that WCS implantation is relatively feasible and efficacious in selected cases; therefore, this approach may be a suitable treatment option for patients with complex vascular diseases of the ICA. The technical success rate, procedural results, and angiographic and clinical follow-up outcomes were consistent with those reported previously (18, 20).

Based on our experience, the advantages of the WCS can be summarized as follows: immediate lesion exclusion from the circulation and vessel wall reconstruction; simple, time-saving, and minimally invasive; low risk of procedure-related rupture or rebleeding because of no/few procedural manipulations in the aneurysmal lumen; and no mass effects or aneurysm shrinkage because of no/minimal application of embolization materials. Despite the advantages mentioned above, the clinical application of the WCS has some limitations, such as the incidence of endoleak, poor target arrival rate when passing the tortuous intracranial vasculature, and side-branch occlusion, which require further discussion.

### Endoleak Management

Endoleak is a common phenomenon during endovascular treatment with covered stents, which implies failure to exclude the disease and carries a high risk of lesion recurrence. Thus, endoleak has become a significant issue after using the covered stent. Generally, endoleak is caused by incomplete occlusion of the disease orifice after covered stent placement, which can be classified into types I–IV according to the source of blood flow (21, 22). Type I endoleak, resulting from blood flow that originates from the proximal or distal endograft attachment sites, is the most common type. Type II endoleaks represent retrograde blood flow through the ICA branch vessels into the aneurysm sac. In this study, seven type I endoleaks and two type II endoleaks were observed.

The different types of endoleak can be treated using the following strategies. First, for type I endoleak, the size of the parent artery should be carefully evaluated to select an adequately sized stent. The inflation pressure should be maintained during deployment until full apposition of the stent is achieved, and negative pressure should be maintained while gently removing the balloon. In addition, tension should be applied if the bending part is at the distal end of the lesion/tension should be reduced if the bending part is at the proximal end of the lesion during release. Also, if endoleak originates from the proximal or distal endograft attachment site, and no contrast agent is retained in the lumen during the later stages, the corresponding endograft attachment site should be dilated with the balloon, or another stent should be implanted. Finally, if retrograde flow endoleak occurs at the distal end of the stent, and contrast agent is retained in the lumen during the later stages, or slow and slight filling of the lumen is observed, follow-up observations should be considered. In this study, three patients had slight endoleak that spontaneously eliminated during follow-up.

Second, for type II endoleak, a microcatheter should be delivered to the branch artery for coil embolization. Based on previous experience of endovascular treatment of thoracic and abdominal aortic aneurysms with covered stents, type II endoleak should be treated immediately to prevent the risk of aneurysmal rupture (23, 24). However, we think that if endoleak blood flow is minimal, follow-up observations should be considered. In this study, one patient underwent embolization of the branch artery, and another patient underwent follow-up observation; endoleak eventually disappeared in both patients.

Third, for type III or IV endoleaks, the catheter “Trojan horse” technique should be immediately applied if the lesion is located beyond the cavernous segment, or if the parent artery is seriously tortuous, which could avoid fabric tears, graft disconnection, or disintegration of the fabric during delivery. Once type III or IV endoleaks appear, immediate implantation of a second covered stent is recommended to prevent the risk of aneurysmal rupture (25).

Based on the above experience, the incidence and outcome of endoleak in the present study were significantly lower compared with previous studies (incidence, 13.8 vs. 30.8%, respectively, outcome, 3.3 vs. 12.8%, respectively) (26).

### Side-Branch Occlusion

Side-branch vessel occlusion is the main disadvantage of WCS treatment as it limits the application of this procedure to certain anatomical locations of the ICA, such as the clinoid–supraclinoid segment. This is the position at which the ophthalmic artery, the posterior communicating artery, and the anterior choroidal artery originate. In our study, side-branch vessel occlusion occurred in four patients, including two cases of ophthalmic artery occlusion, one case of anterior choroidal artery occlusion, and one case of posterior communicating artery occlusion. Before the procedure, the locations of side-branch arteries and aneurysms should be carefully identified from multiple angles. If perforating arteries will inevitably be covered by the stent, the collateral circulation should be evaluated. In this study, before the procedure, the existence of a collateral circulation was verified in patients with high-risk closure of side branches. Therefore, none of the four patients in our study showed acute or delayed clinical symptoms, such as visual impairment, limb weakness, or diplopia after side-branch vessel occlusion.

Although coverage of the side-branch vessel did not cause serious neurological deficiencies in this study, closure of side branches should be avoided as much as possible. Zhu et al. reported acute right-sided visual impairment caused by closure of the right ophthalmic artery with the WCS, which might have been due to insufficient compensation of lateral branches from the external carotid artery (27). Studies have reported that the posterior communicating artery can be considered covered if the vessel is not of the fetal type (28). The anterior choroidal artery is an important side-branch of the ICA, and its occlusion can cause limb palsy, aphemia, and loss of consciousness; hence, coverage of the anterior choroidal artery should be avoided (29).

### In-Stent Stenosis

In-stent stenosis is another concern of WCS placement. One report suggested that the in-stent stenosis rate of covered stents in the coronary circulation is ~30% (30). However, in our series, four patients (6.15%) who underwent angiographic follow-up presented with asymptomatic mild to moderate in-stent stenosis, similar to that reported by Tan et al. (31). We speculated that the relatively low incidence of in-stent stenosis could be ascribed to fewer risk factors for atherosclerosis in patients with dissecting/traumatic aneurysm and CCF. Moreover, a stringent dual antiplatelet therapy regimen after the procedure was another vital factor that reduced the incidence of in-stent stenosis after WCS implantation. It has been demonstrated that dual antiplatelet therapy could inhibit in-stent neointimal hyperplasia by inhibiting platelet aggregation and activation, as well as smooth muscle hyperplasia (32).

## Limitations

This study has some limitations that should be highlighted. First, long-term patient follow-up was not conducted; hence, the long-term patency of the WCS and in-stent stenosis rate remain unclear. Second, our study adopted a retrospective design and lacked a comparative control group of patients undergoing other treatments, such as stent-assisted coil embolization or FDS. Third, the number of patients enrolled was relatively small, so a larger sample size is required to confirm the outcomes.

## Conclusion

Our study revealed that the treatment of patients with complex vascular diseases of the ICA using WCS implantation is relatively safe, feasible, and efficacious. The WCS showed excellent short-term target artery patency and satisfactory clinical outcomes. WCS implantation could be a feasible method for complex vascular diseases of the ICA treatment by lesion isolation and vessel wall reconstruction. Endoleak is the major issue after initial covered stent placement, which can be eliminated or dramatically diminished by repeat balloon dilation and/or placement of an additional covered stent. Nevertheless, the clinical outcomes need to be further confirmed by studies with a longer follow-up period and controlled studies with larger sample sizes.

## Data Availability Statement

The raw data supporting the conclusions of this article will be made available by the authors, without undue reservation.

## Ethics Statement

Written informed consent was obtained from the individual(s) for the publication of any potentially identifiable images or data included in this article.

## Author Contributions

All authors contributed to the study conception and design, commented on previous versions of the manuscript, read, and approved the final manuscript. Material preparation, data collection, and analysis were performed by LM, HF, SY, and J-CX. The first draft of the manuscript was written by LM, H-QT, and CF.

## Conflict of Interest

The authors declare that the research was conducted in the absence of any commercial or financial relationships that could be construed as a potential conflict of interest.
